# A Comprehensive Review on Anxiolytic Effect of *Lavandula Angustifolia* Mill. in Clinical Studies

**DOI:** 10.1002/fsn3.70993

**Published:** 2025-09-22

**Authors:** Sana Manzoor, Allah Rakha, Hina Rasheed, Zuhaib F. Bhat, Muhammad Shaffay Ali Khan, Gholamreza Abdi, Rana Muhammad Aadil

**Affiliations:** ^1^ National Institute of Food Science and Technology University of Agriculture Faisalabad Pakistan; ^2^ Department of Nutrition and Dietetics The University of Faisalabad (TUF) Faisalabad Pakistan; ^3^ Division of Livestock Products Technology SKUAST‐Jammu Jammu India; ^4^ Institute of Home and Food Sciences Government College University Faisalabad Pakistan; ^5^ Department of Biotechnology, Persian Gulf Research Institute Persian Gulf University Bushehr Iran

**Keywords:** anxiety, anxiolytic property, essential oils, lavender, volatile compounds

## Abstract

*Lavandula angustifolia* , a fragrant but bitter flower generally termed lavender, has been exploited for years as a therapy for distinct ailments. It can act as an antispasmodic, analgesic, hypotensive, antiseptic, antimicrobial, and tonic effects that promoting overall health and vitality. This review critically appraises the major bioactive components of lavender and their anxiolytic effects. These key elements interact with the nervous and endocrine systems to modulate the pathophysiology and improve mood and behavior. They modulate the GABA receptors to relax the neurons, spare more serotonin in synapses, and channelize neurotransmitters through distinct signaling cascades. Several clinical trials have demonstrated the anxiolytic potential of oral lavender, with minimal side effects, in human subjects. Therefore, exploiting the herbs for therapeutic purposes, that is, lavender for anxiety, is a safer approach to managing anxiety and preserving mental health and well‐being.

## Graphical abstract

Lavender has been traditionally used as a medicinal herb to relieve stress and anxiety. Linalool and linalyl acetate are the main bioactives, along with terpenes and alcohols. Lavender modulates receptors and neurotransmitters to downregulate the excitation. Neuroplasticity is restored via signaling cascades and calcium channels. Medicinal forms of lavender are being marketed as commercial products.

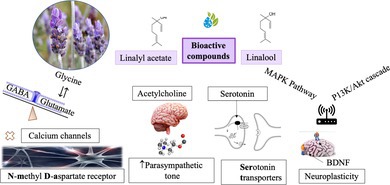

## 1 Introduction

Overall, both animals and humans can benefit from the incorporation of plant extracts into diets, promoting better health outcomes while also emphasizing the need for ongoing research to fully understand their impacts (Abduallah et al. 2023; Al‐Saeed et al. 2023; Bagheri et al. 2024; Gul et al. 2024; Salman et al. 2024; Hammoud et al. 2025). *Lavandula angustifolia* Mill. is a coniferous shrub that is categorized in the Lamiaceae family (WFO 2024). The plant is generally called lavender and has been exploited for centuries for its preventive and curative properties against certain ailments. It possesses a distinct fragrance with a bitter flavor and is enriched with valuable volatile compounds (Uritu et al. 2018). It encompasses about 47 species and is typically a small, evergreen shrub that can grow between 1 and 3 feet tall. They feature gray‐green leaves that are narrow and lanceolate, measuring about 1–2 in. in length. The leaves are aromatic when bruised and can appear silvery due to fine hairs covering them. The flowers of lavender are one of its most distinctive features. They grow on long spikes that can reach lengths of 8–16 in. (20–40 cm) and bloom in various colors, including shades of purple, blue, lilac, and occasionally white or yellow. Each flower spike can hold numerous small blooms that are tubular with five lobes—two on the upper lip and three on the lower lip. The flowering period typically occurs from late spring to mid‐summer (PlantVillage 2024). There are several popular species and cultivars of lavender, for example, English Lavender (known for its sweet fragrance and culinary uses), French Lavender (features unique flower spikes with long petals), Spanish Lavender (characterized by its distinctively shaped flower heads), and Lavandin (a hybrid known for its strong fragrance and oil production) (Wells and Lis‐Balchin 2002).

Essential oils are obtained primarily through steam distillation of the flower spikes to obtain volatile compounds, resulting in a concentrated oil that contains a complex mixture of phytochemicals such as linalool and linalyl acetate. On the other hand, flower extracts are typically made by soaking the plant material in solvents, including non‐volatile components, which can enhance the flavor and aroma profile of the extract. The pharmaceutical industry mostly utilizes high‐ester oils from *L. angustifolia* Mill, whereas lower‐quality oils from other subspecies are commonly used for fragrance purposes (Sarkic and Stappen 2018). Bulgaria takes the lead as the largest producer of lavender essential oils, generating up to 200 MT. According to available statistics, lavender cultivation in Bulgaria covered a total area of 7021 ha in the year 2018. On the other hand, Islamabad, Pakistan, also has a small‐scale lavender cultivation. However, due to its low cultivation and oil production, the small amount that is produced has more waste than significance (Boshnakova‐Petrova 2020). Owing to its versatile utility, lavender is being produced in small gardens at homes as well as large‐scale farms for industrial use (Shahid et al. 2023). Monographs are detailed documents that provide comprehensive information about a substance, including its uses, benefits, risks, and regulatory status. For lavender, several monographs exist. The EMA has published monographs on lavender essential oil, detailing its traditional uses for conditions such as anxiety and sleep disturbances. The U.S. National Center for Complementary and Integrative Health (NCCIH) provides information on the safety and efficacy of lavender, highlighting its uses in aromatherapy and potential side effects. The World Health Organization (WHO) has included lavender in its list of medicinal plants with established traditional uses (Royal Botanic Gardens, Kew 2024). Various countries have their herbal pharmacopeias that include entries on lavender, discussing its historical uses and recommended dosages. Lavender‐based essential oils allow for usage in a variety of other products, such as candles and cleaning solutions, adding a touch of relaxation to any environment. When incorporated into food items, it undoubtedly boosts the nutritional profile, sensory experience, and durability (Wells et al. 2018; Manzoor, Rakha, Rasheed, et al. 2024).

The essential oil, which is present in the oil glands situated between the sebaceous glands and the small hairs on the surface of the calyx, is the primary constituent of lavender. Essential oils are available in a concentration range of 2%–3%. Steam distillation or hydro distillation is often used to extract essential oils from the lavender (Prusinowska and Śmigielski 2014). Linalool and lavandulol, along with their esters, that is, linalool acetate and lavandulol acetate, create the fruity‐fatty fragrance found in fresh flowers owing to the presence of butyl and hexyl esters. Herbal flavors are produced by ketones and monoterpene aldehydes. Sesquiterpenes and derivatives of santalene are responsible for the sweet fragrant aromas (Lawrence 2011; Śmigielski et al. 2013). The most commonly used lavender for therapeutic purposes is known as English lavender, which is enriched with essential oils, anthocyanins, phytosterols, sugars, minerals, coumaric acid, glycolic acid, valeric acid, ursolic acid, herniarins, coumarins, and tannins. Furthermore, they are employed as food additives owing to their peculiar flavors and smells, as well as their antioxidant, antibacterial, antifungal, insecticidal, and insect repellent properties. Lavender originates from a variety of species that are used to flavor foods and baked products (Kakraliya et al. 2022).

Lavender essential oils derived from plants possess a multitude of beneficial properties. These include their ability to relieve muscle spasms and cramps, ease digestive discomfort, alleviate pain, promote relaxation and sleep, lower blood pressure, prevent the growth of microorganisms, kill bacteria and viruses, combat fungal infections, reduce urine production, and provide tonic effects, thus promoting overall health and vitality (Blažeković et al. 2018).

Anxiety is a typical response to extremely stressful circumstances; if left untreated, it could get worse. Over time, anxiety's interference with our emotional, cognitive, and behavioral responses may result in considerable harm (Latreille 2020). It affects one‐third of people in their lifetime, leading to health and functional issues (Bandelow and Michaelis 2022). The global prevalence of depression and anxiety is estimated to be 20.5% and 25.8%, respectively (Johns et al. 2022). According to one meta‐analysis, a significant percentage of university students in Pakistan (43%) suffer from symptoms of depression (Khan et al. 2021). Therefore, the management of anxiety through herbal and medical approaches has been adopted for better outcomes. In the light of the therapeutic properties of lavender, this review endeavors to assess its anxiolytic potential, specifically focusing on the modulation of behavior at the neuroendocrine level, as substantiated by previous clinical studies.

### 1.1 Phytochemical Composition and Biosynthetic Pathways

**FIGURE 1:**
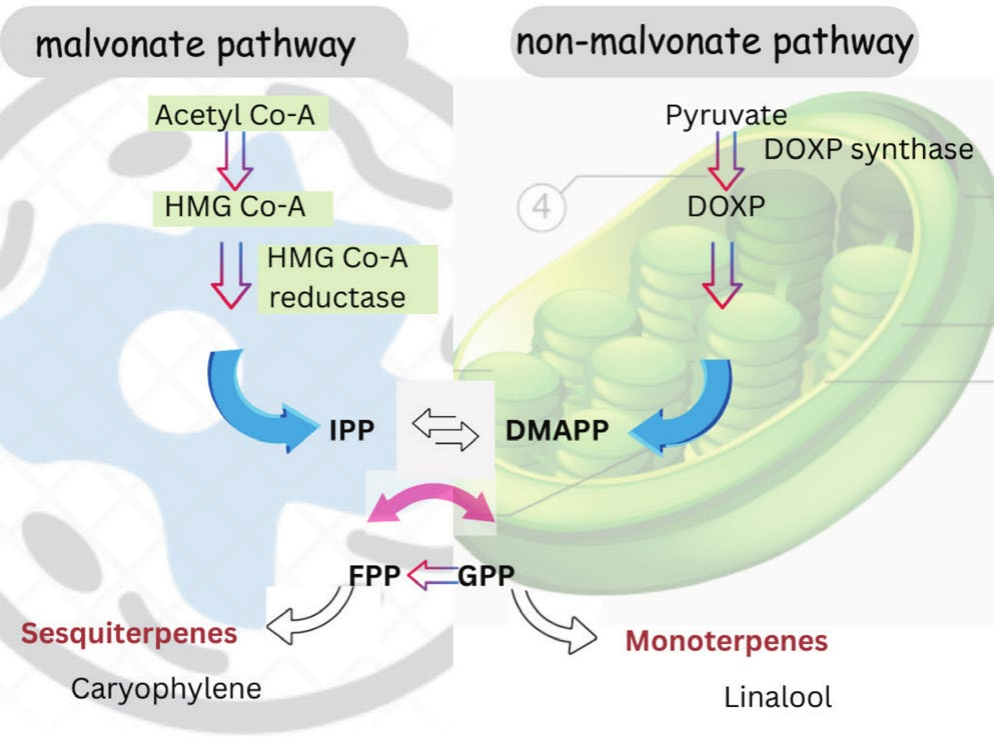
Biosynthesis of active ingredients in lavender.

The essential oil from lavender has a yellow to orange color and a strong odor, with a yield of about 2%. However, the aqueous extract yield is much higher (about 22%) with a dark tone (Slimani et al. 2022). The bioactive components of lavender, including some of the terpenes and ester compounds that are potentially involved in its mechanism and aromatic efficiency, are summarized in Table 1. The constituents of Lavandula essential oils are mainly monoterpenes and sesquiterpenes. These active ingredients are biosynthesized from the C5 units, isopentenyl diphosphate and dimethylallyl diphosphate. Its synthesis starts from acetyl‐CoA to HMG CoA, leading to the mevalonate (MVA) pathway in the cytosol. HMG CoA reductase activity regulates the metabolic flux through the MVA pathway and eventually forms isoprenoid end‐products (Vranová et al. 2013). In plastids, one molecule of pyruvate goes through the non‐mevalonate (MEP) pathway. The MEP pathway can be regulated by several enzymes, including 1‐deoxy‐d‐xylulose 5‐phosphate (DXP) synthase (DXS), DXP reductor‐isomerase (DXR), and hydroxymethyl‐butenyl 4‐diphosphate (HMBPP) reductase (HDR) (Carretero‐Paulet et al. 2002; Mendoza‐Poudereux et al. 2015). One molecule of IPP (isopentenyl pyrophosphate) and one molecule of DMAPP (dimethylallyl pyrophosphate) are condensed to yield geranyl diphosphate (GPP) in both the cytosol and plastids. GPP is the precursor of most of the monoterpenes. It leads to the formation of linalool and limonene. Further reduction of one GPP with one IPP produces farnesyl diphosphate (FPP), the precursor of most of the sesquiterpenes. Hence, there is a wide array of terpenes resulting from the derivatization of GPP or FPP through the incorporation of different terpene synthases as shown in Figure 1 (Chatzopoulou et al. 2003; Lange and Ahkami 2013; Boeckelmann 2008). Other terpenes, such as furanoid linalool oxide and linalool oxide, adding aroma to the extract, have also been found in minor concentrations. The chemical structures of these components are presented in Supporting Information as cdx file format (Aprotosoaie et al. 2017).

**TABLE 1:** Composition of lavender essential oil and aqueous extract.

Compound	Essential oil (%)	Aqueous extract (%)	References
m‐Xylene	0.11 ± 0.00	0.06 ± 0.00	Yuan et al. (2019); Kozuharova et al. (2023); Ciocarlan et al. (2021)
α‐Pinene	3.13 ± 0.03	0.69 ± 0.00	
Camphene	0.96 ± 0.02	0.38 ± 0.00	
β‐Pinene	0.90 ± 0.03	0.31 ± 0.00	
3‐Octanone	0.19 ± 0.00	0.20 ± 0.00	
β‐Myrcene	1.13 ± 0.03	0.10 ± 0.00	
3‐Carene	1.23 ± 0.00	0.37 ± 0.00	
Hexylacetate	0.59 ± 0.00	0.37 ± 0.00	
*m*‐Cymene	1.97 ± 0.02	0.54 ± 0.00	
D‐Limonene	0.53 ± 0.00	0.05 ± 0.00	
1,8‐Cineole	1.13 ± 0.00	2.29 ± 0.01	
γ‐Terpinene	0.11 ± 0.01	0.06 ± 0.00	
Linalool	34.94 ± 0.15	60.87 ± 0.02	
Camphor	0.38 ± 0.00	0.78 ± 0.00	
Borneol	2.52 ± 0.02	1.25 ± 0.00	
Lavandulol	0.21 ± 0.01	0.42 ± 0.01	
Terpinene	1.87 ± 0.00	4.21 ± 0.01	
α‐Terpineol	0.41 ± 0.01	0.85 ± 0.00	
Hexyl butyrate	0.17 ± 0.00	—	
Linalyl anthranilate	42.18 ± 0.25	24.05 ± 0.04	
Lavandulyl anthranilate	0.84 ± 0.00	0.63 ± 0.00	
Linalyl acetate	30.42 ± 4.68	—	
Neryl acetate	2.14 ± 0.02	0.61 ± 0.05	
Caryophyllene	2.17 ± 0.03	—	
cis‐β‐Farnesene	0.13 ± 0.00	—	
Other constituents	1.01 ± 0.85	0.98 ± 0.14	

Various studies have been done to evaluate the chemical composition of lavender (Tomescu et al. 2015; Andrei et al. 2018; Pokajewicz et al. 2021). Secondary metabolites can be released from the plant matrix with the use of an enzyme‐assisted extraction. These techniques are efficient, convenient, and eco‐friendly (Hosni et al. 2013). Some of the extraction procedures are infusion, digestion, percolation, decoction, Soxhlet extraction, and ultrasound‐assisted extractions (Abubakar and Haque 2020). The summarized composition of *L. angustifolia* is given in Table 1 with the percentage composition of each bioactive compound along with its standard deviation (indicated by ±).

### 1.2 Ethnobotanical Uses of Lavender

Lavender has been used for medicinal and aromatic purposes by ancient Greeks, Romans, and Egyptians. It was believed to have antiseptic, calming, anti‐inflammatory, and antioxidative properties, making it popular during the Middle Ages. Herbal medicines containing lavender oil are considered effective, although there is not enough evidence from clinical trials. These supplements have been safely used for at least 30 years, and medical supervision is not required for their intended uses (Products n.d.). It has been over 30 years since lavender oil and flowers were introduced in the EU, but it cannot be suggested that lavender flowers or oil is a well‐established treatment for general anxiety disorders. Despite the improvement in the quality of studies over time, the number of patients treated with essential oil of lavender in randomized controlled trials (RCTs) is still insufficient. (Kasper et al. 2010; Kasper et al. 2014; Kasper et al. 2016).

The ancient Greeks would use lavender to fight insomnia. A 2015 study of 158 postnatal mothers found that the lavender fragrance helped improve their quality of sleep significantly. Another study of Japanese students found that exposure to the aroma of lavender oil during the night improved the students' ability to wake up the next morning (Yogi et al. 2021). The EU established a long‐standing use and tradition of lavender flowers, where they mention the possible effect for the relief of mild symptoms of mental stress, exhaustion, and to aid sleep (Afshar et al. 2015).

For thousands of years, the genus Lavandula's essential oils and plant extracts have been utilized to treat a wide range of illnesses. The chemical makeup of the two most widely used species of lavender, i.e., *L. angustifolia* Mill. and Lavandula intermedia Emeric ex Loisel., has been extensively researched (Rai et al. 2020). The majority of the work has been focused on the oil's antifungal properties, despite the fact that *L. angustifolia* oil and one of its primary components, linalool, have been proven to have antibacterial action against *Escherichia coli* and *Salmonella* spp. etc. (Hosni et al. 2013; Van Dyk and Pletschke 2012). Volatile essential oil extracts have been utilized therapeutically for a very long time. Aromatherapy is considered a promising treatment for its psychological and physical advantages of breathed volatile molecules (Cui et al. 2022). It is believed that the limbic system, particularly the amygdala and hippocampus, mediates the further consequences (Masuo et al. 2021). The phenolic compounds of lavender, particularly gallic and rosmarinic acids, provide a pleasant, calming scent. Moreover, its mydriatic, antispasmodic, anticholinergic, analgesic, and calming properties have all been extensively studied. Lavender was once used to treat Parkinson's disease, stomach ulcers, and asthma in traditional medicine (Kim and Cho 1999).

### 1.3 Neuroprotective and Anxiolytic Potential

The essential oil from lavender helps to reduce the degradation of acetylcholine while simultaneously increasing the levels of superoxide dismutase (SOD), catalase (CAT), and glutathione by inhibiting acetylcholinesterase (AChE). It has the potential to reduce oxidative stress and prevent memory loss. Moreover, the aqueous fraction is considered to lower neuron decay mediated by lipid peroxidation via decreased malondialdehyde (MDA) levels (Mushtaq et al. 2018a, 2018b). It also affects the cholinergic system of neurons and reverses the impaired ones due to the presence of linalool (Hritcu et al. 2012). Besides special memory enhancement, it also decreases the glutamate‐induced toxicity in neurons that can cause cell damage and ultimate death (Kashani et al. 2011). Amyloid β protein plaques from the brain were also eliminated efficiently in animal trials. These plaques may also lead to Alzheimer's disease (Soheili et al. 2012). The levels of acetylcholinesterase and malondialdehyde have been found to decrease, whereas antioxidant enzymes such as superoxide dismutase and glutathione peroxidase have been shown to increase, potentially protecting PC12 cells against H_2_O_2_ (Xu et al. 2016).

In an in vitro study, PC12 cell culture was used for analysis. The neurotoxic effects of A1‐42 protein and its components were observed against lavender and its components. A1‐42 protein plays a critical role in the development of Alzheimer's disease. The research showed that the pro‐apoptotic enzyme caspase‐3 synthesis could be inhibited through various morphological deviations and ROS in nerve cells. This inhibition is done by counteracting the neurotoxic effects of A1‐42 protein components and dysregulation of calcium channels. Voltage‐gated calcium channels (VGCC) and N‐methyl‐D‐aspartic acid receptors are modulated to hinder the neurotoxic effects. Consequently, they further regulate the Ca^2+^ channels to reduce oxidation and activate caspase‐3, setting off an apoptotic cascade (Caputo et al. 2016). In another in vitro investigation, the antioxidant, antiaggregative, and antiacetylcholinesterase potentials of lavender were witnessed (Simsek et al. 2021). Lavender, having a significant antioxidant potential without any cytotoxicity, can be utilized in the treatment of neurodegenerative diseases such as Alzheimer's. N‐methyl‐D‐aspartic acid receptors are regulated along with extracellular repositioning to decrease the neurotoxic effect of glutamate (Dobson and Giovannoni 2019).

The increased function of acetylcholinesterase has been significantly protected by linalool, and the lowered activity of glutathione peroxidase and superoxide dismutase was likewise protected by the chemical malondialdehyde. Additionally, they considerably preserved the expression of heme oxygenase‐1 and nuclear factor erythroid. Its pharmacological therapy resulted in decreased expression of proteins involved in synaptic plasticity, brain‐derived neurotrophic factor, p‐CaMKII, and calcium‐calmodulin‐dependent protein kinase II (CaMKII). Overall, the expression of proteins involved in the nuclear factor erythroid 2‐related factor 2/heme oxygenase‐1 pathway, cholinergic activity, oxidative stress, and synaptic plasticity may be protected by lavender essential oil and linalool. Linalool and lavender essential oil might therefore be thought of as potential agents for enhancing cognitive impairment in neurological illnesses (Xu et al. 2016). The following are the mechanisms of anxiolytic potentials attributed to lavender.

Rather than directly inducing sleep, indirect anxiolytic effects have been demonstrated in many clinical studies of anxiety disorders (Kasper et al. 2018; Seifritz et al. 2019). It appeared to lack any inherent sedative or sleep‐inducing qualities as compared to the most sedative anxiolytics, including benzodiazepines and pregabalin, which have a propensity for abuse and dependence (Schifano 2014; Manzoor, Rakha, Altemimi, et al. 2024).

Linalool (28.47%) and linalyl acetate (38.36%) are the major ingredients responsible for anxiolytic potential (Li et al. 2016; Xu et al. 2023). It has been observed that linalyl acetate possesses the ability to inhibit calcium channels and modulate NMDA receptors. These actions ultimately lead to the activation of the inhibitory pathway and a reduction in neuronal excitability. On the other hand, linalool has been found to increase extracellular serotonin levels by blocking serotonin transporters. This mechanism of action results in a reduction in anxiety and an improvement in overall well‐being (López et al. 2017; Khatri et al. 2020). According to the research conducted by Araj‐Khodaei et al. (2020), it has been established that the sedative properties of the medication may cause drowsiness in certain patients.

#### 1.3.1 Alteration of Voltage‐Dependent Calcium Channels

Various studies have shown alterations in the functioning of calcium channels. As evidenced by an experiment, unreasonably high doses of hypnotic drugs mainly attack GABAergic and glutamatergic receptor systems, as well as several neurotransmitter transporters. These targets also encompass the primary pharmacological characteristics associated with other minor tranquilizers (López et al. 2017). A medication named Silexan is purely derived from lavender and is termed its extract. This medicine is often prescribed for anxiety and holds the status of over‐the‐counter medicine in Germany. The effect of Silexan was checked for calcium influx induced by depolarization. Potassium channels were also associated with ion release in synaptosomes of the mouse's brain. The potential action of the test drug was solely attributed to linalyl acetate and linalyl cinnamate, as other terpenes and alcohols did not show any change. Serotonergic pathways appear to be crucial (Chioca et al. 2013). This is consistent with earlier work where Silexan raised extracellular levels of serotonin and dopamine in the mouse brain, as shown by microdialysis trials. These neurotransmitters are mood stabilizers and modulate the behavior of humans (Baldinger et al. 2015). Essential oils have been observed to have an impact on the lipid bilayer by inhibiting the transmission of neurotransmitters and limiting the influx of ions (Karan 2019).

#### 1.3.2 Neuroplasticity

Neuroplasticity is defined as the ability of the human nervous system to rearrange and mend itself in response to any external or internal stimuli, which may be an injury to the brain or stroke. Amendments in structure and functioning are done in this regenerative ability (Puderbaugh and Emmady 2022). Almost all the antidepressant medications are solely attributed to neuroplasticity for key pathways to exhibit the desired mechanism of action, and it takes place concurrently with the amelioration of depressive and anxious symptoms (Harmer et al. 2017). This is consistent with previous findings that suggest a key role for impaired structural and functional neuroplasticity in the pathophysiology of depression (Levy et al. 2018). Traditional antidepressants are thought to counteract some fluctuations by increasing neuronal and synaptic plasticity, which is evidenced by some receptors and signaling cascades. They are associated with monoaminergic receptors and the conforming postsynaptic signal cascades, which get activated and stimulate the brain for target functioning (Duric and Duman 2013). Brain‐derived neurotrophic factor, that is, BDNF, is an endogenous activator of synaptogenesis in primary neurons. Confocal microscopy was used to examine the effects of treatment on synaptogenesis in the neurons present in the hippocampus of rats (Friedland et al. 2021). Even at the lowest antianxiety medication dosages (0.1 g/mL/kg body weight), the number of synapses increased; however, it was less pronounced than the outcome of BDNF. In an experimental study, inhaling lavender oil available in the market increased the growth of neurons in the supraventricular region and hippocampus of mice treated with corticosterone. Moreover, the complexity of dendrites was increased in the hippocampus as compared to control groups treated with corticosterone, but immature neuronal cells showed no modulation in response to treatment (Sánchez‐Vidaña et al. 2019). As evidenced by another experimental study, corticosterone‐treated rats' serum had lower levels of BDNF. In addition, the designed treatment containing lavender enhanced the quantity of hippocampal immature neural cells and serum BDNF levels in non‐corticosterone‐treated control rats (Sánchez‐Vidaña et al. 2019).

#### 1.3.3 Activating Intracellular Signaling Cascade

Numerous signaling pathways, including the phosphoinositide 3‐kinase (PI3K)/protein kinase B (Akt) cascade, the mitogen‐activated protein kinase (MAPK) pathway, and the cAMP‐mediated phosphorylation of protein kinase (PKA), play their roles in the development of neurons and synaptic plasticity. Studies have shown that the flavonoids of lavender and its other bioactive compounds have a potential influence on these pathways. The MAPK pathway disseminates, amplifies, and integrates information to initiate appropriate physiological responses, including cell proliferation, cell differentiation, growth, inflammatory responses, and demise in mammalian cells from a variety of stimuli. Long‐term memory function, brain development, and the responses to linalyl acetate and linalool have all been linked to MAP kinase signaling (Amini et al. 2023). Many phosphokinases are involved in behavioral psychology as their activities are stimulated by various neurotransmitters that are involved in alertness, mood control, and anxiety (Wang et al. 2021). These pathways altogether combine to phosphorylate cAMP Response Element Binding Protein (CREB) (Heiser et al. 2013). Then, they bind to the cAMP response element (CRE), thus controlling the transcription of downstream substrates that control the survival and expression of various transcription factors and growth factors, as well as metabolic activities in neurons (Wang et al. 2018). Research studies have shown that the pharmacological property of Silexan is attributed to its bioactive profile for activating these intracellular signaling pathways. It is a proprietary essential oil derived from *L. angustifolia* and manufactured by steam distillation of flowers (Müller et al. 2021).

#### 1.3.4 Modulation of Receptors and Neurotransmitters

Serotonin transporters located in the synaptic cleft regulate extracellular serotonin in the synapse region. The active ingredient of lavender, that is, linalool, blocks these receptors, thereby increasing the amount of serotonin available at the synapses. Serotonin transporters are inhibited to spare more serotonin to stabilize mood and behavior. N‐methyl D‐aspartate receptors are also antagonized by linalyl acetate by blocking calcium channels and thus enhancing gamma‐aminobutyric acid (GABA) action to counterbalance stimulatory glutamate. These mechanisms of controlling receptors, in turn, exert an inhibitory effect and reduce anxiety (López et al. 2017; Khatri et al. 2020). Neurotransmitters are also regulated by lavender. Linalool inhibits acetylcholine release from vesicles and drains into the synaptic cleft, thus relaxing the smooth muscles of the arteries. This inhibition decreases blood pressure and other metabolic processes that are energy‐demanding to calm down the nervous system. The ultimate goal of such alterations is to switch the sympathetic tone of the nervous system to a parasympathetic one (Magden et al. 2023).

### 1.4 Clinical Studies of Lavender as Anxiolytic

Oral lavender can be used in the form of being incorporated into food products and medicines, etc. However, the most common and traditional use of lavender is aromatherapy, in which dilutions are made and inhaled for a short time through a ventilator or spraying method. Massage with lavender oil can also exhibit its anxiolytic action via topical application. Additionally, it has been demonstrated that massage can boost vagal activity by applying pressure, lower cortisol levels, foster the development of immunity, and alleviate stress (Field 2014; Manzoor et al. 2025). Additionally, the rubbing motion utilized during the massage aids in the absorption of the aromatherapy essential oils and reveals their therapeutic impact. Additionally, it is believed that by exposing the fragrances, the person can breathe in the pleasant odors and that this aids in their recovery (Buckle 2015). The processing of olfactory memory takes place within the hippocampus, which is situated in the limbic system. It is a well‐established fact that encountering a pleasant odor that is familiar has a positive impact on an individual's well‐being (Steflitsch and Steflitsch 2008). The following are some clinical studies to justify the anxiolytic potential of lavender as it has been used in human efficacy trials via aromatherapy and oral intake as well.

In an experiment done by Kasper et al. (2015), patients with anxiety‐related restlessness and disrupted sleep were studied to determine the relaxing impact of lavender. The potential ingredients exerted a significant impact on anxious behavior. Another experiment was done to characterize lavender for its chemical constituents, and the level of anxiety of patients was evaluated according to Spielberger's questionnaire, which indicated a significant reduction in stress levels. The therapeutic effect of lavender was attributed to monoterpenes present in its bioactive profile (Bakhsha et al. 2014).

Bazrafshan et al. (2020) performed an experiment to determine how lavender tea affects older individuals' mean levels of depression, trace anxiety, and state anxiety. Due to its affordability and accessibility, lavender herbal tea is recommended for usage as a supplemental treatment to lessen anxiety as well as depression in light of the study's findings that it can lower depression and anxiety scores.

Seifritz et al. (2019) conducted a clinical study to estimate if the improvement in sleep quality attributed to lavender extract is due to its primary anxiolytic effect or to its secondary sedative effect. For this purpose, oral administration of 80 mg drug per day was given for 10 weeks. According to the findings, lavender extract almost solely improved secondary sleep through its anxiolytic impact rather than sedation. The results supported the drug's claimed mode of action.

In another trial, a cotton ball with 10% lavender essential oil was utilized as an intervention. After following 15 min of being instructed to smell the cotton ball, the participants had a significant difference in anxiety score (*p* < 0.05), indicating its anxiolytic potential in pain and stress conditions (Abbaszadeh et al. 2020).

Iranian students often struggle with test anxiety. In a study by Bekhradi and Vakilian (2016), they sought to determine how lavender aromatherapy affected exam anxiety. However, the intervention group had more anxiety‐free kids than the control group (*p* = 0.03). The results revealed that using lavender in aromatherapy could boost the proportion of pupils who are anxiety‐free.

It can also be seen that patients with other diseases experiencing anxiety may get an advantage by using lavender oil. In a study, patients who had type II diabetes mellitus (DM) along with insomnia were given inhaled lavender and a placebo for 4 weeks twice a day. In the early morning and evening, the Pittsburgh Insomnia Rating Scale‐20 (PIRS‐20), WHOQOL‐BREF, and Beck Depression Inventory (BDI) indicated that inhaled lavender had significantly improved mental health (Lari et al. 2020).

Addressing dental anxiety is one of the most difficult duties for pediatric dental professionals. Due to the dangers associated with these procedures, aromatherapy substantially decreases dental anxiety. In an experimental study, children's anxiety levels were assessed using the incorrect Backer scale and provided aromatherapy of lavender. The treatment group demonstrated a substantial shift in post‐treatment anxiety levels in relation to the Wong Packer Pain Scale, as well as a reduction in pulse rate and breathing rate, as compared to controls (Abd Alkhalek 2023).

In an open and controlled randomized trial conducted by Hasanzadeh et al. (2016), lavender essential oil inhalation was assessed for its impact on anxiety due to pain experienced during chest tube removal, tested to ascertain the effects of cold application. In comparison to the cold application group, there was a considerably lower anxiety score owing to lavender oil.

The goal of a study by Franco et al. (2016) was to ascertain whether giving ladies lavender flower oil (LFO) aromatherapy before having breast surgery would lessen their anxiety or not. This study compared the effects of LFO and unscented oil (UO) on a single location. It could be concluded that the positive results were brought about by the given treatment in terms of aromatherapy along with the patients receiving more care.

The goal of the study was to assess how lavender cream, with or without a footbath, affected pregnant women's tension, anxiety, and depressive symptoms. At the end of the study, it was demonstrated that rather than lavender and footbath, lavender alone led to lower levels of anxiety, tension, and sadness than the placebo group. By employing such techniques, pregnant women may live more comfortably and function more effectively during this crucial time as explained through the experiment done by Effati‐Daryani et al. (2015).

Farshbaf‐Khalili et al. (2018) conducted a trial in which they found the association of lavender and bitter orange with anxiety in women during the period of postmenopause. Results indicated that administering 500 mg of lavender twice daily for 8 weeks was beneficial in reducing anxiety in postmenopausal women. Due to their moderate and temporary adverse effects, lavender also did not offer any significant health risks.

After an intervention of preoperative aromatherapy with lavender oil, it can be stated that lavender inhalation minimizes anxiety levels and improves sleep quality in individuals undergoing colorectal operations. Lavender oil massage may be advised in the run‐up to surgery to lower anxiety, improve sleep quality, and boost patient satisfaction (Ayik and Özden 2018).

A randomized clinical trial was performed by Tugut et al. (2017) to compare the levels of anxiety in women who had gynecological exams both before and after a consultation and who received lavender oil aromatherapy or a placebo. The Visual Analogue Scale score for pain interpretation fell together with the state anxiety score in the intervention group.

An investigation observed how hemodialysis patients' levels of anxiety and sadness were affected by aromatherapy using lavender essential oil. ANOVA evaluated a significant reduction in depressive symptoms before and after enough time of using the intervention. Hence, various concentrations can be used to relieve anxiety in clinical situations regarding operations and therapies to relax the subjects and control the consequences of depression and anxiety (Bagheri‐Nesami et al. 2017).

In a comprehensive review, the anxiolytic efficacy of various lavender administration routes was analyzed. Inhalation enables rapid absorption through the pulmonary epithelium, resulting in a quicker onset of action and bypassing first‐pass metabolism (Dong and Zhuang 2024). During inhalation, volatile compounds of lavender such as linalool and linalyl acetate channelize the limbic system via the olfactory nerve, thereby modulating GABAergic neurotransmission to exert anxiolytic and sedative actions. This method has proven to reduce anxiety and stress‐related symptoms (Buch and Von Fraunhofer 2019). It is a non‐invasive method with a quick process and minimal systemic side effects. While its effects are observed only for a short time, and depend upon the concentration and efficacy of active ingredients. Oral administration involves gastrointestinal absorption and hepatic first‐pass metabolism, resulting in reduced bioavailability and increased risk of systemic drug interactions. The oral route is mostly adopted in pharmacotherapy where systemic circulation is involved. Systemic absorption allows the active substances to have central nervous system (CNS) effects via potential neurogenesis driven by GABAergic modulation. Its effectiveness is suited for prolonged effects. Prolonged systemic retention of the drug increases the potential for pharmacokinetic and pharmacodynamic interactions. Furthermore, the delayed onset of action renders this route less effective when compared to topical administration (Shakya et al. 2018; Sánchez‐Vidaña et al. 2019; El‐Tokhy et al. 2023). Aromatherapy, a form of inhalation using volatile essential oils, exerts its effects primarily through olfactory stimulation and limbic system modulation, offering mild, short‐term psychological and physiological benefits. Aromatherapy is mostly preferred for short‐duration treatments as compared to other oral dosages. Massage therapy also seemed efficacious in various clinical trials. Lavender oil gets absorbed in minutes after inhalation, achieving a peak plasma level in 60–90 min (Hur et al. 2014). Anxiolytic effects have been demonstrated across diverse settings, including preoperative care, where it may reduce anxiety without the side effects of benzodiazepines. Aromatherapy offers a rapid onset, while oral lavender (Silexan 80 mg/day) shows superior long‐term efficacy. Meta‐analyses support its significant anxiety‐releaving impact over placebo and comparable efficacy to lorazepam, but without sedation or withdrawal. Lavender aromatherapy is, clinically, the best route of administration for short‐duration, while Silexan (oral lavender) 80 mg is preferable for long‐term treatment of anxiety. Clinical evidence is presented in Table 2

**TABLE 2:** Clinical studies showing the anxiolytic effect of lavender.

References	Subjects	Route of delivery	Duration	Methodology	Tool for assessment	Findings
Abd Alkhalek (2023)	Children from outpatient dental clinic	Inhalation	Once	*n* = 20 Group 1: 5 drops of LEO for 3 min Group 2: 5 drops of orange essential oil (OEO) Group 3: Control	Wong Packer Pain Scale Pulse rate Respiratory rate Oxygen saturation	↑ Oxygen saturation ↓ Wong Packer Pain Scale, pulse, and respiratory rate
Abbaszadeh et al. (2020)	Hospitalized patients for bone marrow biopsy	Inhalation	Once	*n* = 40 Group 1: Control Group 2: 3 drops of 10% LEO for 15 min	Visual Anxiety Scale (VAS)	↓ Anxiety Score (*p* < 0.001)
Bazrafshan et al. (2020)	Elderly subjects	Oral	Twice per day for 4 weeks	*n* = 30 Group 1: Control Group 2: 2 g lavender tea bag	Beck Depression Inventory (BDI) and Spiel Berger Anxiety Inventory	↓ BDI and anxiety inventory (*p* < 0.001)
Lari et al. (2020)	Patients with type 2 diabetes	Inhalation	4 weeks	*n* = 18 Group 1: Control Group 2: 3 drops of LEO for 5 min	Pittsburgh Insomnia Rating Scale (PIRS) WHO Quality of Life (WHOQOL) Beck Depression Inventory (BDI)	↓ PIRS, WHOQOL and BDI (*p* < 0.01, *p* < 0.01 and *p* < 0.04)
Ayik and Özden (2018)	Hospitalized patients for colorectal surgery	Massage	Twice	*n* = 40 Group 1: Control Group 2: 5% LEO for 10 min	State Anxiety Inventory (SAI) and Richard‐Campbell Sleep Questionnaire (RCSQ)	↓ STAI and RCSQ (*p* < 0.05)
Farshbaf‐Khalili et al. (2018)	Post menopausal women	Oral	8 weeks	*n* = 40 Group 1: Control Group 2: 500 mg lavender powdered extract Group 3: 500 mg bitter orange powdered extract	State anxiety I Trait anxiety II	↓ State and trait anxiety (*p* < 0.01 and *p* < 0.04)
Bagheri‐Nesami et al. (2017)	Hospitalized patients for hemodialysis	Inhalation	4 weeks	*n* = 38 Group 1: Control Group 2: 3 drops of 5% LEO for 10 min	Hamilton Anxiety and Depression Scale (HADS)	↓ HADS (*p* < 0.005)
Tugut et al. (2017)	Outpatients in gynecology clinic	Inhalation	Once	*n* = 35 Group 1: Control Group 2: 3 drops of 10% LEO for 10 min	VAS STAI	↓ VAS and STAI (*p* < 0.005 and *p* < 0.005)
Bekhradi and Vakilian (2016)	Adults	Inhalation	7 days	*n* = 45–65 Group 1: Control Group 2: 5–6 drops of LEO	Test anxiety scale	↓ Anxiety scale (*p* < 0.03)
Hasanzadeh et al. (2016)	Patients having a chest tube after coronary artery bypass grafting (CABG)	Inhalation	Once	*n* = 20 Group 1: Control Group 2: cold gel pack Group 3: 1–2 drops of LEO for 20 min Group 4: cold gel pack and LEO	VAS Short form and modified‐McGill pain questionnaire (SFM‐MPQ) Spielberger situational anxiety level inventory (STAII)	↓ VAS, SFM‐MPQ and STAII (*p* < 0.001) in Groups 2 and 3
Franco et al. (2016)	Female to undergo breast surgery	Inhalation	Once	*n* = 47 Group 1: Control Group 2: 2 drops of 2% lavender fleur oil (LFO) for 10 min Group 3: 2 drops of 2% unscented oil (UO) for 10 min	STAI	↓ STAI (*p* < 0.001) in Group 2
Effati‐Daryani et al. (2015)	Pregnant women (2nd trimester)	Topical	8 weeks	*n* = 47 Group 1: Control Group 2: 2 g lavender cream and footbath Group 3: 2 g lavender cream	DASS‐21	↓ Anxiety (*p* < 0.05) in Groups 2 and 3
Kasper et al. (2015)	Adults	Oral	10 weeks	*n* = 74 Group 1: Control Group 2: 8o mg LEO	HAM‐A	↓ Anxiety (*p* < 0.03)
Bakhsha et al. (2014)	Hospitalized patients for Curettage	Inhalation	Once	*n* = 50 Group 1: Control Group 2: LEO for 1 min	Spielberger's State Anxiety Inventory	↓ Anxiety (*p* < 0.0001)

### 1.5 Toxicity

Lavender oil can be toxic if ingested in large amounts. Symptoms of poisoning may include nausea, vomiting, diarrhea, difficulty breathing, and confusion. Ingestion of approximately 5 mL (0.17 fl oz) of diluted essential oil can cause toxicity in adults; for children, as little as 2–3 mL (0.068–0.101 fl oz) may be toxic (Therapeutic Guidelines 2021). Instances of toxicity from lavender use are exceedingly rare. Laboratory experiments (in vitro) and animal studies (in vivo) demonstrate that LEO exhibits no cytotoxic effects. Furthermore, oral administration of LEO is safe, with an LD_50_ (lethal dose required to kill 50% of a test population) of 13.5 g/kg. It is worth noting that prolonged exposure to linalool, a compound found in lavender oil, may elicit allergic reactions. As a result, the 7th Amendment of the European Cosmetics Legislation mandates that natural products containing linalool be labeled as potentially allergenic (Cardia et al. 2018).

In an in vitro experimental study, a non‐toxic concentration of lavender has been termed as 0.5–100 μg/mL. Mutagenic activity induced when reaching the toxic concentration is attributed to linalyl acetate (Di Sotto et al. 2011). In another trial, the approximate median lethal oral dose was determined to be greater than 2000 mg/kg (Mekonnen et al. 2019). According to the Globally Harmonized Classification System for Chemical Substances and Mixtures of acute systemic toxicity, the *L. angustifolia* EO was assigned class 5 status (2000 mg/kg), which has been considered as the lowest toxicity class (Wilhelm and Maibach 2012). The finding of this toxicity assessment was aligned with the outcomes of a research trial conducted by Silva et al. (2015).

## 2 Conclusion

Lavender has been used traditionally for therapeutic purposes attributed to its remarkable volatile components, particularly linalool and linalyl acetate. It is potentially active in improving behavior and restoring mental health. The anxiolytic effect of lavender is owed to its key ingredients, that is, linalool and linalyl acetate. It interferes with the neuroendocrine system and metabolic pathways to modulate the nervous system, as evidenced by clinical trials described in the current review. Therefore, the utilization of lavender to relieve anxiety is the best way to deal with the side effects with a safe and economical approach. However, despite its therapeutic promise, the current body of research lacks sufficient consistency in dosage, administration methods, and study designs. These gaps raise chief concerns about the reproducibility and generalizability of clinical findings. Therefore, while lavender shows substantial potential as a complementary intervention for anxiety, it should not yet be considered a standalone solution. Although it is advisable that further high‐quality trials are required to be conducted to homogenize the study and comprehend safety concerns. Therefore, lavender can be integrated into therapeutic practices with evidence‐based outcomes of its significance and limitations.

## Author Contributions

**Sana Manzoor:** conceptualization (equal), writing – original draft (equal). **Gholamreza Abdi:** writing – original draft (equal), writing – review and editing (equal). **Allah Rakha:** validation (equal), writing – original draft (equal), writing – review and editing (equal). **Zuhaib F. Bhat:** data curation (equal), software (equal), writing – original draft (equal), writing – review and editing (equal). **Hina Rasheed:** review and editing. **Muhammad Shaffay Ali Khan:** resources (equal), writing – original draft (equal). **Rana Muhammad Aadil:** conceptualization (equal), investigation (equal), writing – original draft (equal), writing – review and editing (equal).

## Conflicts of Interest

The authors declare no conflicts of interest.

## Supporting information


**Data S1:** fsn370993‐sup‐0001‐DataS1.docx.

## Data Availability

Data will be available on request from the first and corresponding authors.
