# Supplementary material for: A Comprehensive Review on Anxiolytic Effect of Lavandula Angustifolia Mill. in Clinical Studies
Source: Food Sci Nutr. 2025 Sep 22;13(9):e70993. doi: 10.1002/fsn3.70993 (PMC12454915; doi:10.1002/fsn3.70993)
Supplement: Supplementary file 1 — Data S1: fsn370993‐sup‐0001‐DataS1.docx. [file FSN3-13-e70993-s001.docx]

**Chemical structure of bioactive compounds in *Lavandula angustifolia***
